# Supplementary material for: Deep biogeographic barriers explain divergent global vertebrate communities
Source: Nat Commun. 2024 Mar 28;15:2457. doi: 10.1038/s41467-024-46757-z (PMC10978928; doi:10.1038/s41467-024-46757-z)
Supplement: Supplementary file 1 — Supplementary Information [file 41467_2024_46757_MOESM1_ESM.pdf]

Supplementary Information

Deep biogeographic barriers explain divergent global vertebrate communities

Peter J. Williams[1,2,3]*, Elise F. Zipkin[1,2], Jedediah F. Brodie[3,4,5]

[1]Ecology, Evolution, and Behavior Program, Michigan State University, East Lansing, MI 48824, USA

[2]Department of Integrative Biology, Michigan State University, East Lansing, MI 48824, USA

[3]Division of Biological Sciences, University of Montana, Missoula, MT 59812, USA

[4]Wildlife Biology Program, University of Montana, 32 Campus Drive, Missoula, MT 59812, USA.

[5]Institute of Biodiversity and Environmental Conservation, Universiti Malaysia Sarawak, 94300 Kota Samarahan, Malaysia.

*Corresponding author: peter.j.williams.110@gmail.com

Supplementary Table 1: Functional traits used, and phylogenetic signal of these traits. Blomberg's K is a metric of phylogenetic signal for continuous traits where 0 indicates no phylogenetic signal (complete randomness) and 1 indicates phylogenetic signal consistent with Brownian motion. Blomberg's K is shown for diet traits and bird foraging strata, where data are percentages used by a species, and for log body mass. Fritz & Purvis' D statistic is a similar metric of phylogenetic signal for binary traits. The D statistic, rescaled to allow comparison with K, is shown for mammal foraging strata, a categorical trait. Statistical significance was calculated by using randomization tests with 1,000 permutations for each trait. All traits have significant phylogenetic signal (K or D > 0), but some traits are relatively evolutionarily labile (K or D < 1), while other traits are phylogenetically conserved (K or D > 1).

Functional trait		Mammal	Bird
Diet	*Fish*	0.049 (p = 0.004)	0.657 (p < 0.001)
	Fruit	0.339 (p < 0.001)	0.511 (p < 0.001)
	Other plant parts	0.541 (p < 0.001)	0.969 (p < 0.001)
	Invertebrates	1.053 (p < 0.001)	0.949 (p < 0.001)
	Nectar	0.116 (p < 0.001)	1.744 (p < 0.001)
	Carrion	0.491 (p < 0.001)	0.798 (p < 0.001)
	Seeds	0.302 (p < 0.001)	0.632 (p < 0.001)
	Vertebrates	0.192 (p < 0.001)	0.809 (p < 0.001)
Foraging stratum	*Aerial*	1.345 (p < 0.001)	0.645 (p < 0.001)
	Aquatic	-	1.505 (p < 0.001)
	Arboreal	1.190 (p < 0.001)	-
	Canopy	-	0.496 (p < 0.001)
	Ground	1.202 (p < 0.001)	0.772 (p < 0.001)
	Scansorial	0.970 (p < 0.001)	-
	Understory	-	0.260 (p < 0.001)
Log body mass		0.547 (p < 0.001)	7.929 (p < 0.001)

Supplementary Table 2: Additional variables besides climate and biogeographic isolation. For each variable, a model was run with that particular variable and the climate variables. We then compared the results to a model that only included climate. Variables that explained >1% of additional variance in comparison to the climate-only model, indicated by (*), were included in final analyses. We ran models on species richness (SR) and functional richness (FR).

	Increase in R^2 compared to climate-only model					
	Bird		Mammal		Bat	
	SR	FR	SR	FR	SR	FR
*Landmass area	2.10%	0.67%	3.26%	1.85%	2.02%	0.92%
*Mean elevation	3.50%	2.62%	7.23%	2.85%	2.03%	0.62%
*Elevational range	4.20%	2.54%	4.57%	0.87%	2.17%	0.54%
Climatic distance, Present vs. Last Glacial Maximum	0.74%	0.46%	0.87%	0.04%	0.63%	0.24%
Climatic distance, Present vs. Mid-Holocene	<0.01%	0.25%	<0.01%	<0.01%	0.02%	0.03%
Past ice cover	0.83%	0.41%	0.99%	0.01%	0.75%	0.04%
Years since significant land conversion	0.05%	0.08%	<0.01%	0.04%	0.03%	<0.01%
Human Impact Index	<0.01%	0.39%	0.03%	0.04%	0.12%	1.69%

Supplementary Table 3: Species richness of bird and mammal orders by realm. Each order shown represents >1% of all bird or mammal species present in the dataset used in this study. Some species occur in multiple realms, so the total species richness across realms may be greater than the total species number for the order. Note that the dataset used in this study excludes pelagic birds, marine mammals, most small islands, and introduced species.

Class	Order	Total species number	Afrotropical	Australian	Madagascan	Nearctic	Neotropical	Oceania	Oriental	Palearctic	Panamanian	Saharo-Arabian	Sino-Japanese
Bird	Passeriformes	5817	1245	350	70	503	2032	332	1293	601	1083	496	790
	Piciformes	472	99			42	214		100	23	97	34	46
	Apodiformes	442	21	4	5	56	289	18	31	8	207	11	12
	Psittaciformes	327	21	61	3	18	144	49	32		59	4	9
	Galliformes	288	55	7	3	32	61	9	91	40	45	29	61
	Charadriiformes	274	97	72	29	69	78	41	98	124	50	83	73
	Accipitriformes	237	75	18	11	43	64	22	75	48	59	61	56
	Columbiformes	232	31	25	4	18	46	43	76	14	45	23	26
	Strigiformes	180	34	12	6	32	44	9	57	25	43	29	31
	Anseriformes	162	30	26	10	49	42	10	46	67	33	36	47
	Gruiformes	145	31	19	12	20	54	18	33	25	28	12	25
	Coraciiformes	143	46	12	9	9	13	24	52	12	20	16	20
	Cuculiformes	134	26	14	15	11	22	19	55	9	22	18	22
	Caprimulgiformes	110	23	6	2	12	41	6	23	6	35	12	7
	Pelecaniformes	108	35	20	18	22	36	19	39	26	34	27	30
Mammal	Rodentia	2080	376	53	26	310	577	81	327	301	194	164	167
	Chiroptera	1059	218	74	48	114	269	77	287	86	228	95	133
	Eulipotyphla	423	153		1	59	10		93	88	33	40	75
	Primates	405	93		95	1	129		78	2	18	6	20
	Carnivora	251	77		9	44	46		84	62	35	59	66
	Cetartiodactyla	227	90			13	21		70	49	10	37	47
	Diprotodontia	123		78				50	2				
	Didelphimorphia	97				7	93				24		
	Lagomorpha	88	12			25	3		17	40	7	14	26
	Dasyuromorphia	72		57				16					
	Afrosoricida	54	24		30								

Supplementary Table 4: Contemporary climate explains diversity as well as or better than past climate. Results shown for linear models of bird and mammal species richness (SR) or functional richness (FR). Each model included climate data from one of three time periods: the present, the Last Glacial Maximum, and the mid-Holocene. When non-contemporary climates had higher R^2 values, they only increased the variance explained by < 1%. As such, we used only present-day climate in all analyses.

Taxon	Metric	Climate period	R^2 for model containing these variables	
			Climate, elevation, area	Climate, elevation, area, biogeographic isolation
Bird	SR	Present	80.2%	82.6%
		Last Glacial Maximum	79.2%	81.3%
		Mid-Holocene	80.3%	82.5%
Bird	FR	Present	85.8%	88.5%
		Last Glacial Maximum	79.6%	85.5%
		Mid-Holocene	84.8%	88.1%
Mammal	SR	Present	80.0%	84.5%
		Last Glacial Maximum	79.9%	85.3%
		Mid-Holocene	80.3%	84.2%
Mammal	FR	Present	82.8%	87.1%
		Last Glacial Maximum	81.5%	86.7%
		Mid-Holocene	82.5%	86.3%

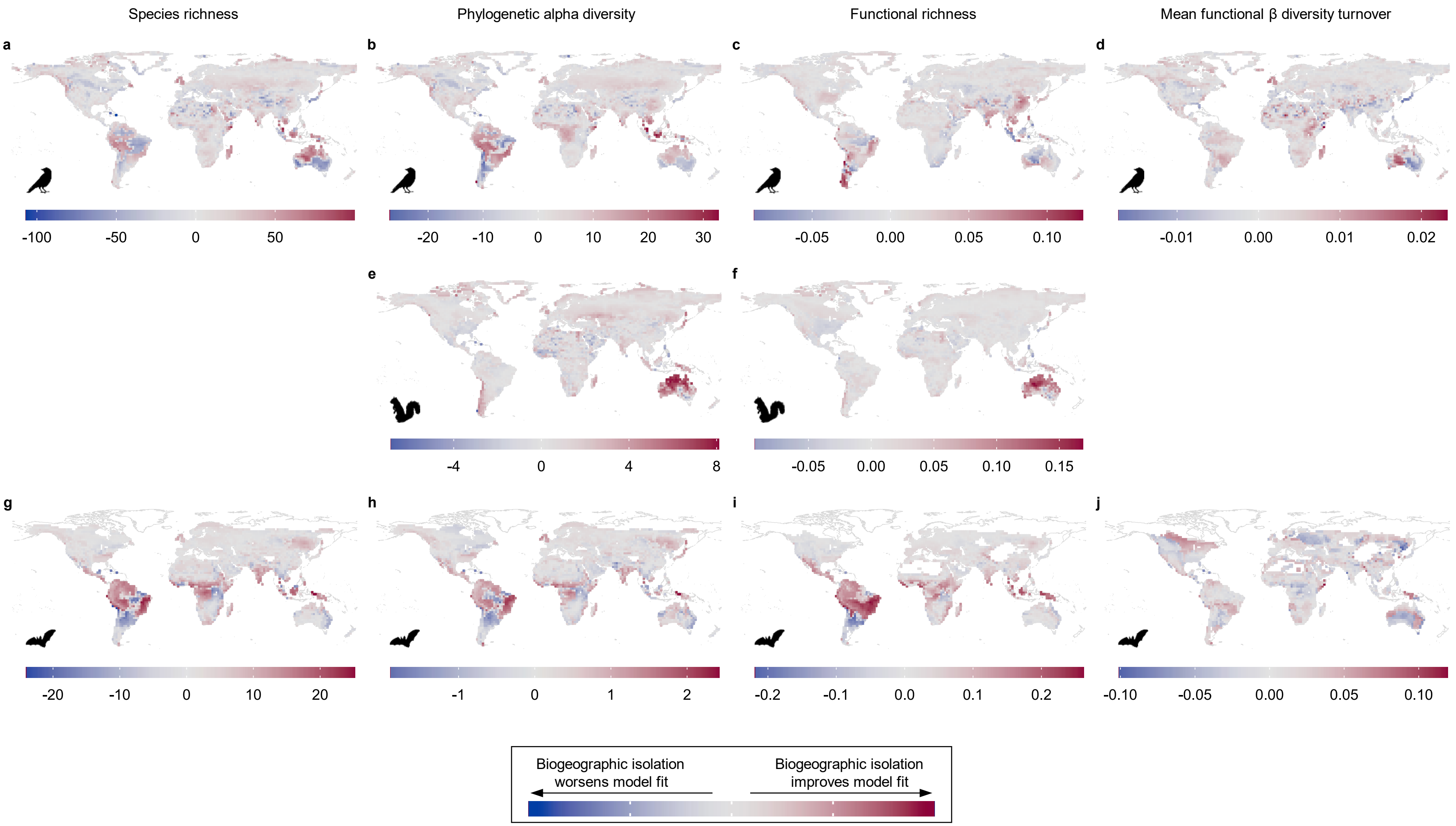

Supplementary Fig. 1: Differences in residuals in models with vs. without biogeographic isolation. Differences in residuals were calculated as the absolute value of residuals in the environment-only model minus the absolute value of residuals in the global model using the residuals values of each grid cell. Scales differ for each plot based on the units of the response variable. Positive values indicate that including biogeographic isolation improved model fit for the grid cells, while negative values indicate that including biogeographic isolation worsened model fit. For birds (a-d), biogeographic isolation did not consistently improve model fit for any regions. For mammals (e-f), biogeographic isolation improved model fit for regions such as Australia. Maps for mammal species richness and mean functional beta diversity turnover are shown in Fig. 3 of the main text. For bats (g-j), biogeographic isolation improved model fit across tropical forest regions, at least for alpha diversity metrics (g-i). Maps are shown with an equirectangular projection.

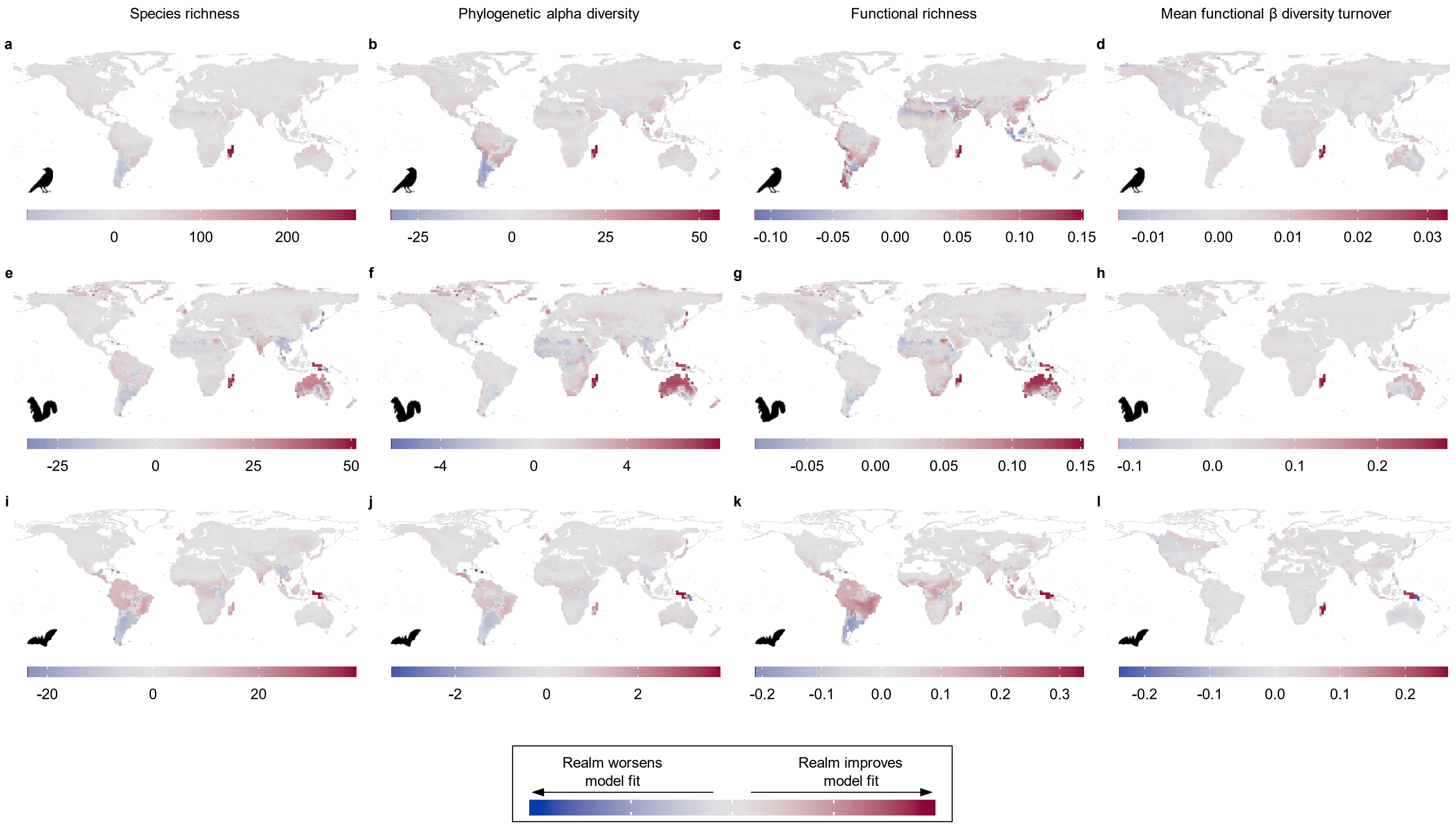

Supplementary Fig. 2: Differences in residuals in models with vs. without realm as a categorical variable. See legend for Supplementary Fig. 1. All plots are analogous to Supplementary Fig. 1, except for plots e & h, which are analogous to Fig. 3 of the main text. In all cases, models were ran using 'realm' as a discrete, categorical variable, rather than using continuous biogeographic isolation quantified using phylobetadiversity. Realms from Holt et al.[1].

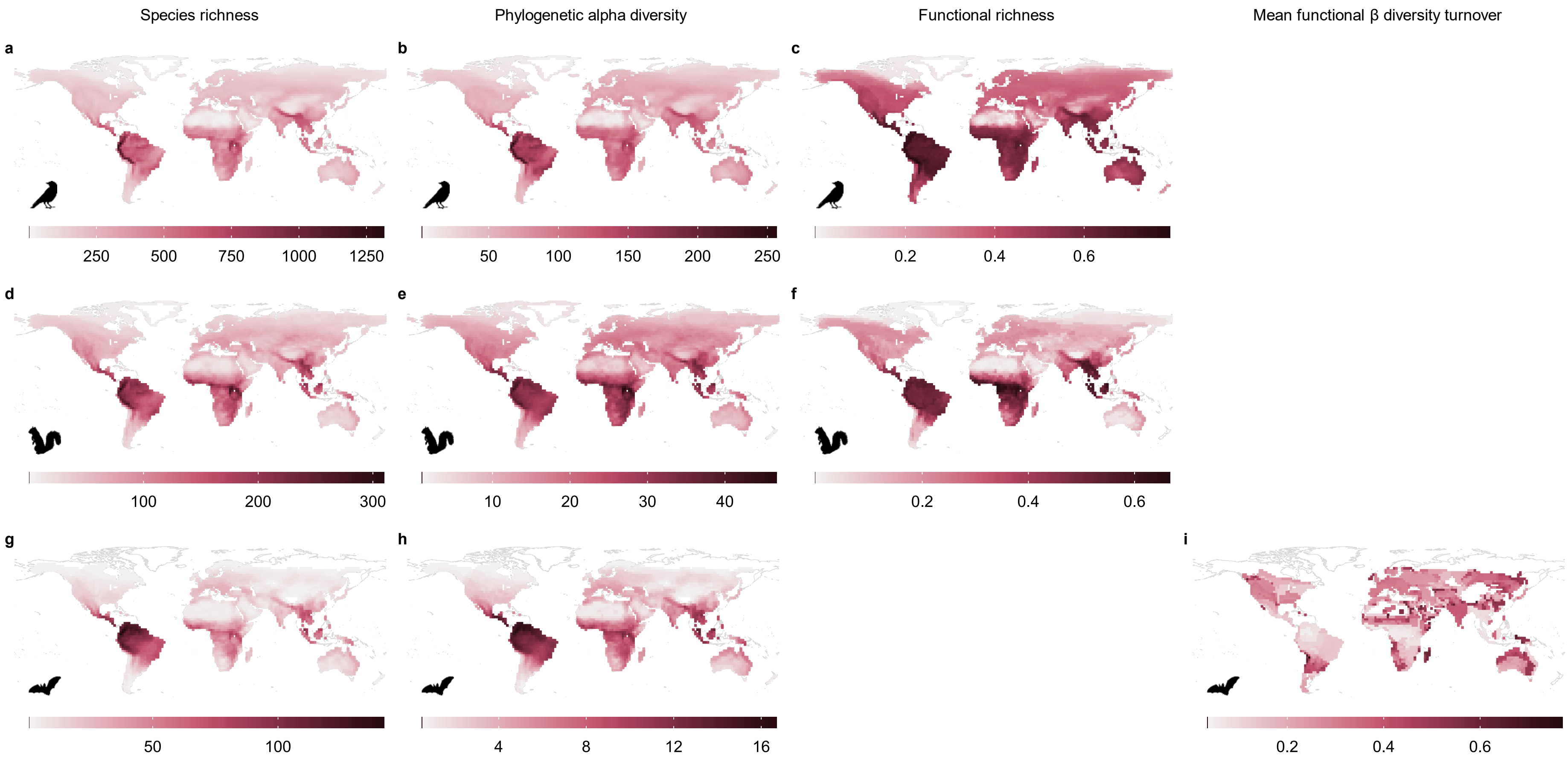

Supplementary Fig. 3: Global biodiversity patterns for (a-c) birds, (d-f) mammals, and (g-i) bats. Bird and mammal mean functional beta diversity turnover are shown in Fig. 4 of the main text. Bat functional richness is shown in Fig. 6 of the main text. Maps are shown with an equirectangular projection.

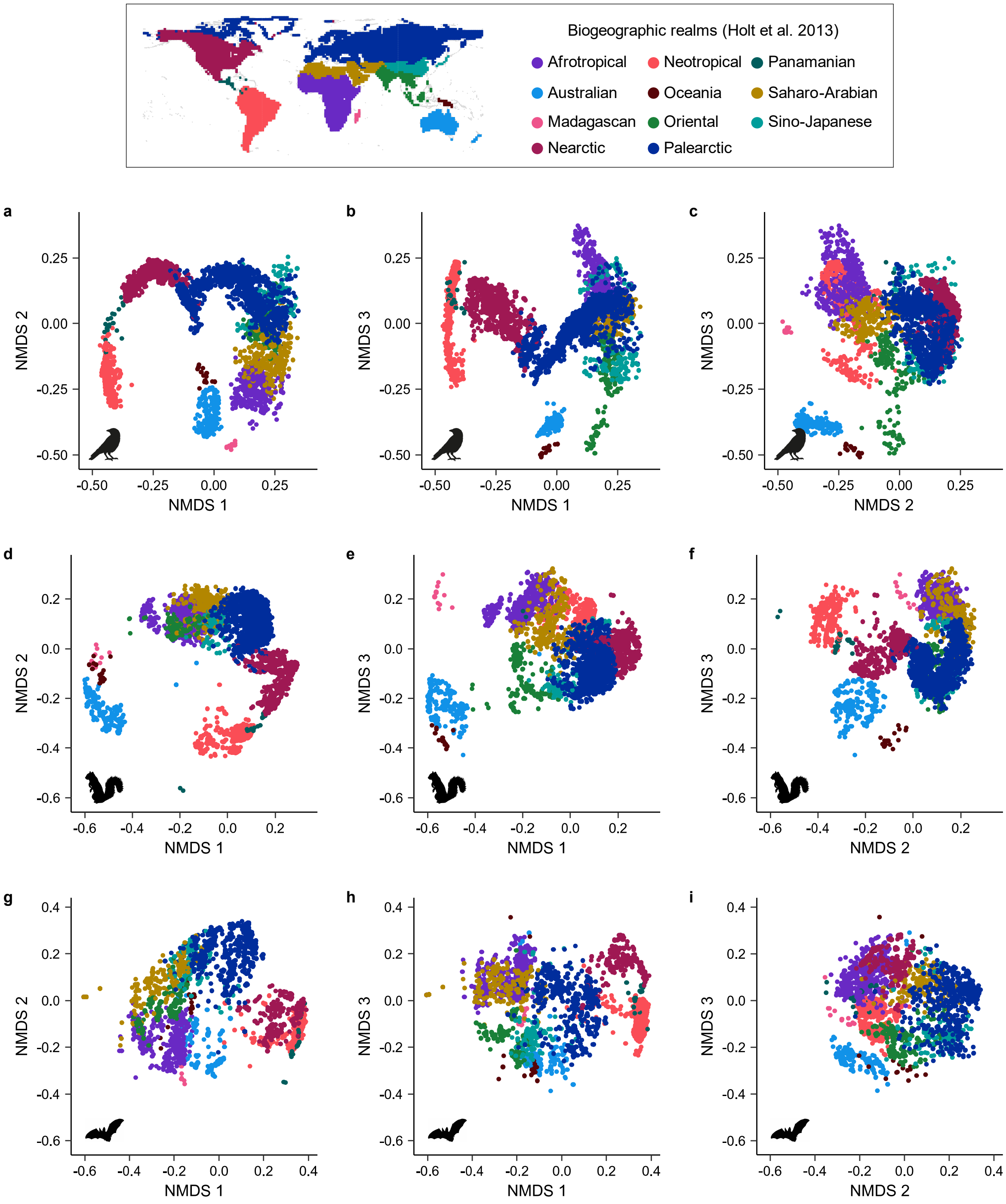

Supplementary Fig. 4: Biogeographic isolation as quantified by phylobetadiversity, with NMDS plots in three dimensions. Phylobetadiversity is shown for (a-c) birds, (d-f) mammals, and (g-i) bats with points colored by biogeographic realm, as categorized by Holt et al.[1]. Plots g and h highlight the biogeographic rift between the Neotropics (Neotropical, Panamanian) and Paleotropics (Afrotropics, Oriental) for bats. The NMDS axes shown here differ from the axes shown in Fig. 1 of the main text, where NMDS were fitted using only two axes (for visualization purposes only). These three NMDS axes were used in analyses as ‘biogeographic isolation’ (stress values 0.156 for mammals, 0.140 for birds, 0.166 for bats).

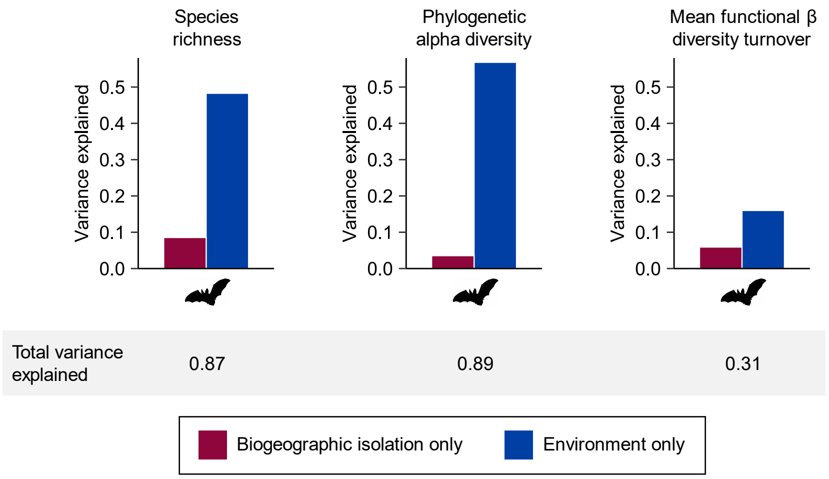

Supplementary Fig. 5: Bat variance partitioning results. In contrast to functional richness (Fig. 6 of the main text), environment explains much more variance than biogeographic isolation for species richness, phylogenetic alpha diversity, and mean functional beta diversity turnover. For each metric, total variance explained includes the variance explained by biogeographic isolation only (red), environment only (including climate, elevation, topography, and landmass area variables; blue), and shared between biogeographic isolation and environment (not shown). Variance explained was calculated by comparing adjusted R^2 values among a biogeographic isolation model, an environment model, and a global model that included both biogeographic isolation and environment variables.

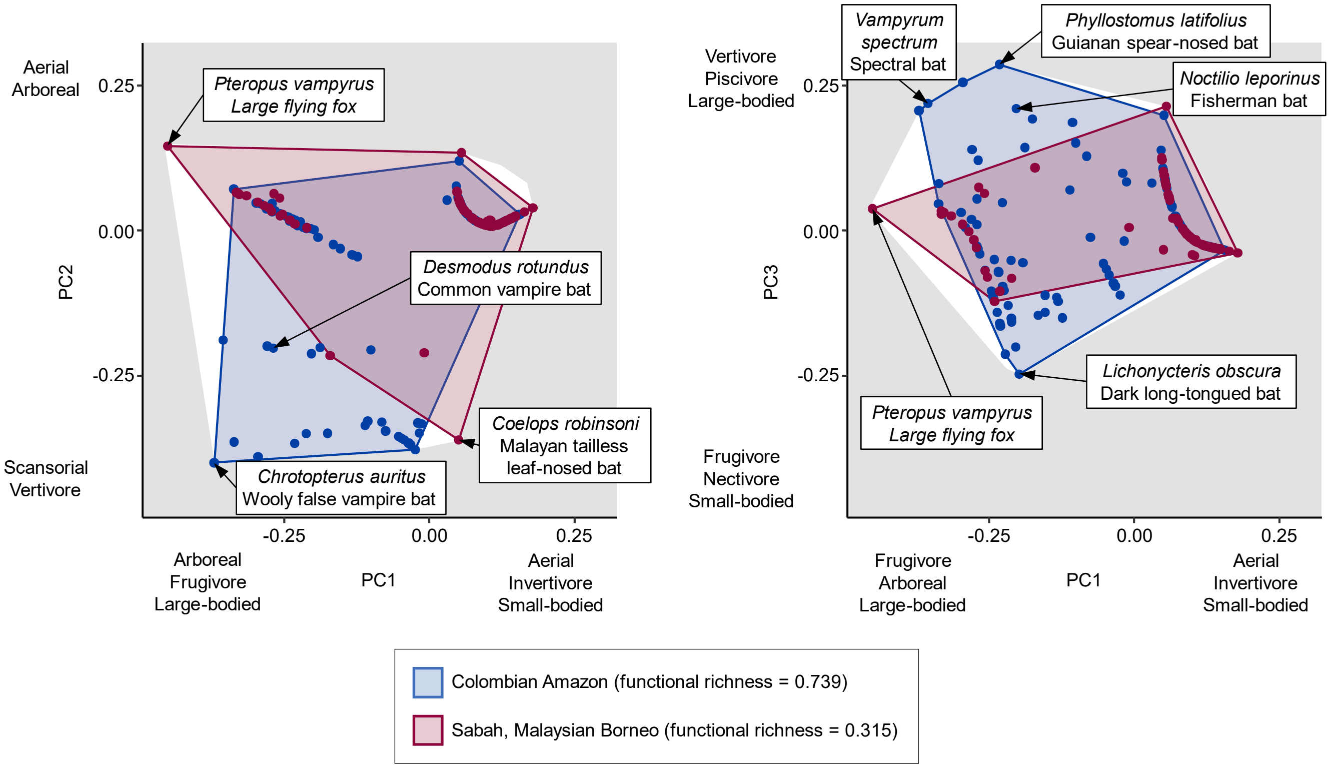

Supplementary Fig. 6: High functional richness in a Neotropical bat assemblage compared to a Paleotropical bat assemblage. The three PCoA axes shown were calculated using trait data from all bat species. Functional richness is the proportion of total functional space occupied by an assemblage, where the white polygons represent the total functional space of all bat species. In this example, the Colombian assemblage contains many vertivorous and piscivorous species which the Malaysian assemblage lacks.

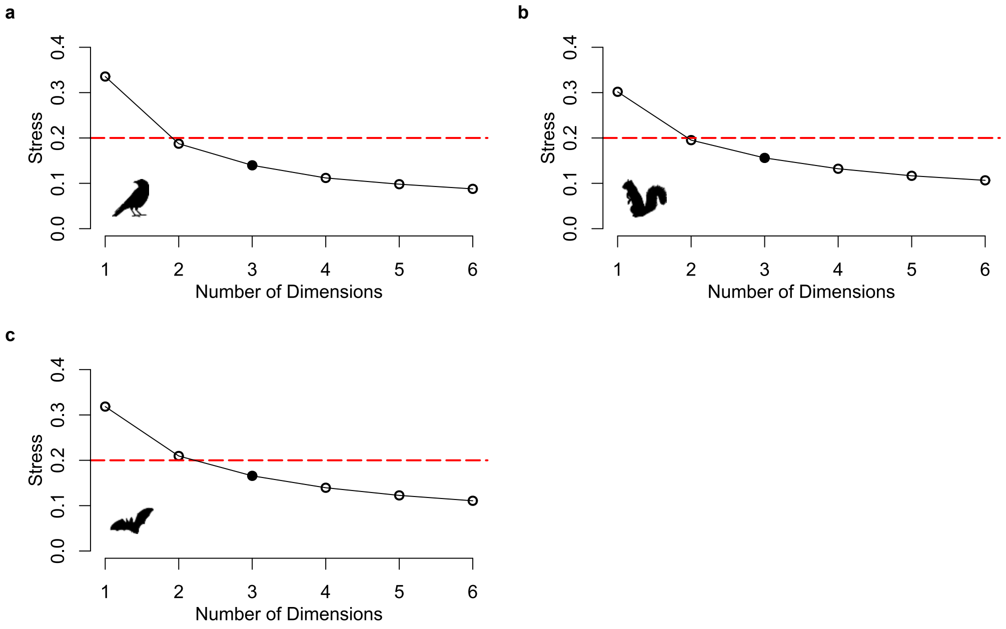

Supplementary Fig. 7: Phylobetadiversity NMDS scree plots for (a) birds, (b) mammals, and (c) bats. According to commonly used guidelines, stress values < 0.2 (the red dashed line) indicate that the NMDS is an acceptable representation of distances, while stress values < 0.1 indicate a very good representation. Based on these scree plots and desire for parsimony in our models, we used three NMDS dimensions (filled circles) in all analyses to represent biogeographic isolation.

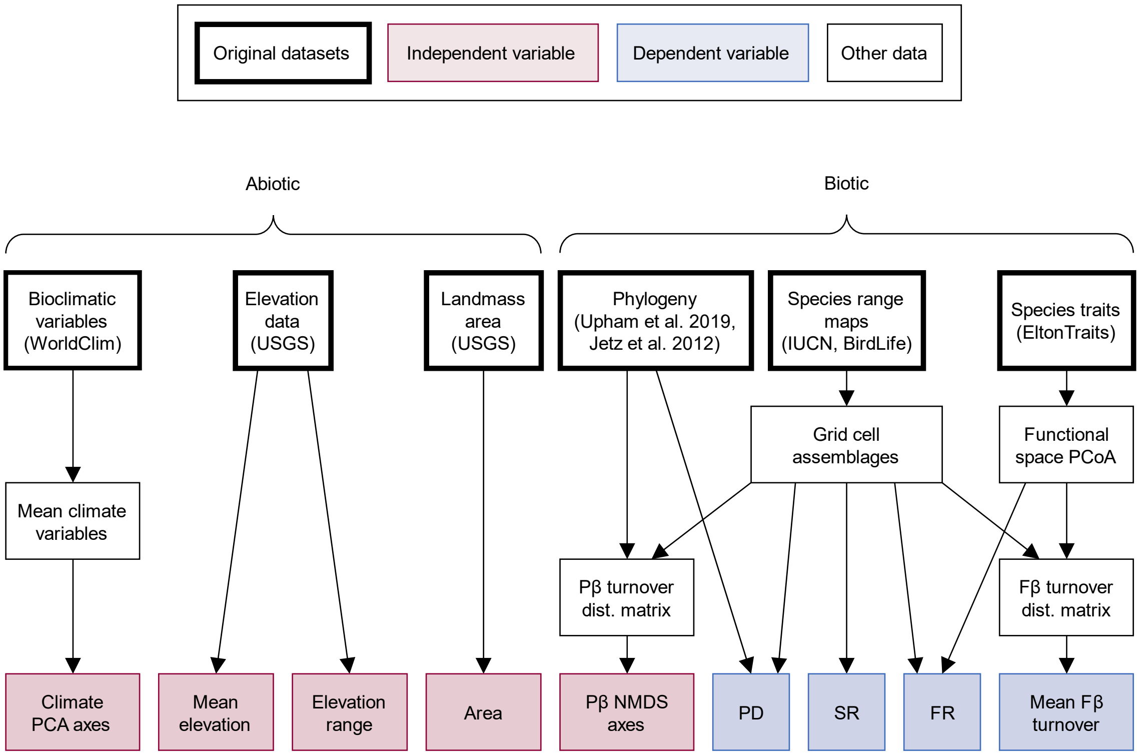

Supplementary Fig. 8: Schematic of methods, deriving variables used in analyses from original data products. Publicly available data products were used to calculate variables for each 2° grid cell. Biotic variables were recalculated separately for each taxon (birds, mammals, bats; see Methods). For each dependent variable (phylogenetic alpha diversity (PD), species richness (SR), functional richness (FR), mean functional beta (Fβ) diversity turnover), we used linear regression including all independent variables shown. In linear regressions, 'environment' variables included four climate PCA axes with quadratic terms, mean elevation with a quadratic term, elevation range, and log landmass area. 'Biogeographic isolation' variables included three phylobetadiversity (Pβ) NMDS axes with quadratic terms and pairwise interactions among axes (see Methods). We then used variance partitioning to calculate the variance explained by biogeographic isolation only, environment only, and shared between biogeographic isolation and environment.

Supplementary References

1. Holt, B. G. *et al.* An update of Wallace's zoogeographic regions of the world. *Science* **339**, 74–78 (2013).
