# Supplementary material for: Deep biogeographic barriers explain divergent global vertebrate communities
Source: Nat Commun. 2024 Mar 28;15:2457. doi: 10.1038/s41467-024-46757-z (PMC10978928; doi:10.1038/s41467-024-46757-z)
Supplement: Supplementary file 3 — Reporting Summary [file 41467_2024_46757_MOESM3_ESM.pdf]

nature portfolio

Corresponding author(s): Peter Jeffrey Williams

Last updated by author(s): Mar 4, 2024

Reporting Summary

Nature Portfolio wishes to improve the reproducibility of the work that we publish. This form provides structure for consistency and transparency in reporting. For further information on Nature Portfolio policies, see our Editorial Policies and the Editorial Policy Checklist.

Statistics

For all statistical analyses, confirm that the following items are present in the figure legend, table legend, main text, or Methods section.

n/a	Confirmed	
☐	☒	The exact sample size (n) for each experimental group/condition, given as a discrete number and unit of measurement
☒	☐	A statement on whether measurements were taken from distinct samples or whether the same sample was measured repeatedly
☐	☒	The statistical test(s) used AND whether they are one- or two-sided<br>*Only common tests should be described solely by name; describe more complex techniques in the Methods section.*
☐	☒	A description of all covariates tested
☐	☒	A description of any assumptions or corrections, such as tests of normality and adjustment for multiple comparisons
☐	☒	A full description of the statistical parameters including central tendency (e.g. means) or other basic estimates (e.g. regression coefficient) AND variation (e.g. standard deviation) or associated estimates of uncertainty (e.g. confidence intervals)
☐	☒	For null hypothesis testing, the test statistic (e.g. F, t, r) with confidence intervals, effect sizes, degrees of freedom and P value noted<br>*Give P values as exact values whenever suitable.*
☒	☐	For Bayesian analysis, information on the choice of priors and Markov chain Monte Carlo settings
☒	☐	For hierarchical and complex designs, identification of the appropriate level for tests and full reporting of outcomes
☒	☐	Estimates of effect sizes (e.g. Cohen's d, Pearson's r), indicating how they were calculated

Our web collection on statistics for biologists contains articles on many of the points above.

Software and code

Policy information about availability of computer code

Data collection	All data processing was performed in R version 4.2.1, except for calculating phylobetadiversity and functional beta diversity, which were performed in R version 4.0.2. The 'betapart' package version 1.5.4 was used to calculate phylobetadiversity, and the mFD' package version 1.0.1 was used to calculate functional beta diversity. The 'stats' package version 4.2.1 was used to calculate PCA. The 'vegan' package version 2.6-4 was used to calculate NMDS. The 'picante' package version 1.8.2 was used to calculate phylogenetic alpha diversity. The 'mFD' package version 1.0.5. was used to calculate functional richness. All code for data processing and analysis are available on GitHub (https://github.com/pwilliams0/Biogeography_and_global_diversity) and Zenodo (https://doi.org/10.5281/zenodo.10779125).
Data analysis	All analyses were performed in R version 4.2.1. The 'stats' package version 4.2.1 was used to run linear models. The 'phytools' package version 1.9-16 and the 'caper' package version 1.0.3 were used to calculate phylogenetic signal. All code for data processing and analysis are available on GitHub (https://github.com/pwilliams0/Biogeography_and_global_diversity) and Zenodo (https://doi.org/10.5281/zenodo.10779125).

For manuscripts utilizing custom algorithms or software that are central to the research but not yet described in published literature, software must be made available to editors and reviewers. We strongly encourage code deposition in a community repository (e.g. GitHub). See the Nature Portfolio guidelines for submitting code & software for further information.

Data

Policy information about availability of data

All manuscripts must include a data availability statement. This statement should provide the following information, where applicable:

- Accession codes, unique identifiers, or web links for publicly available datasets
- A description of any restrictions on data availability
- For clinical datasets or third party data, please ensure that the statement adheres to our policy

Bird range map data are available from BirdLife (http://datazone.birdlife.org/species/requestdis), and mammal range map data are available from the IUCN RedList (https://www.iucnredlist.org/resources/spatial-data-download); we used range map data downloaded in 2018. Species trait data are available from EltonTraits (https://doi.org/10.6084/m9.figshare.c.3306933.v1). Phylogenies for birds and mammals are available from VertLife (https://vertlife.org/data/). Climate data are available from WorldClim (https://www.worldclim.org/data/bioclim.html). Elevation data are available from the United States Geological Survey (https://www.usgs.gov/coastal-changes-and-impacts/gmted2010). Landmass area data are available through the United States Geological Survey's Global Island Explorer (https://rmgsc.cr.usgs.gov/gie/gie.shtml). Derived data products, including grid cell alpha diversity values and pairwise phylobetadiversity, are available on GitHub (https://github.com/pwilliams0/Biogeography_and_global_diversity) and Zenodo (https://doi.org/10.5281/zenodo.10779125).

Research involving human participants, their data, or biological material

Policy information about studies with human participants or human data. See also policy information about sex, gender (identity/presentation), and sexual orientation and race, ethnicity and racism.

Reporting on sex and gender	N/A
Reporting on race, ethnicity, or other socially relevant groupings	N/A
Population characteristics	N/A
Recruitment	N/A
Ethics oversight	N/A

Note that full information on the approval of the study protocol must also be provided in the manuscript.

Field-specific reporting

Please select the one below that is the best fit for your research. If you are not sure, read the appropriate sections before making your selection.

☐ Life sciences ☐ Behavioural & social sciences ☒ Ecological, evolutionary & environmental sciences

For a reference copy of the document with all sections, see nature.com/documents/nr-reporting-summary-flat.pdf

Ecological, evolutionary & environmental sciences study design

All studies must disclose on these points even when the disclosure is negative.

Study description	We used IUCN and BirdLife range maps to define bird and mammal assemblages in 2x2 degree latitude-longitude grid cells. For these grid cells, we calculated alpha diversity metrics including species richness, phylogenetic diversity, and functional richness; beta diversity metrics including phylobetadiversity turnover and functional beta diversity turnover; and environmental metrics including climate, elevation, and landmass area. All of these metrics were derived from publicly available data products.
Research sample	Our research sample includes all bird species (excluding pelagic birds) and all mammal species (excluding marine mammals).
Sampling strategy	We used 2x2 degree latitude-longitude grid cells, keeping cells that covered >50% land, resulting in 3718 grid cells. We did not use finer-scale grid cells due to the computational difficulties in calculating pairwise beta diversity metrics.
Data collection	All data used in the study were publicly available, including range map data, phylogenies, trait data, climate data, and other environmental data. We indicate in the main text and our data availability statement the sources where we downloaded these data.
Timing and spatial scale	All data products used are on a global scale. Range map data from the IUCN and BirdLife were downloaded in 2018. Other data products have not been updated since we downloaded them. Refer to the cited sources for when these data products were originally created.
Data exclusions	We excluded marine mammals, pelagic birds, and portions of ranges where species were introduced or considered vagrant or transitory migrants. We excluded these species and these ranges to better understand natural (non-anthropogentic) dynamics of terrestrial systems.

Reproducibility	Raw datasets and data processing code are available on GitHub and Zenodo so that others may reproduce how we derived the data used in our analyses from original publicly available data products.
Randomization	We did not have experimental treatments, so we did not use randomization. Our study used full global datasets, and our analyses are deterministic.
Blinding	We did not use blinding in our study, as we had no participants and no experimental treatments.

Did the study involve field work? ☐ Yes ☒ No

Reporting for specific materials, systems and methods

We require information from authors about some types of materials, experimental systems and methods used in many studies. Here, indicate whether each material, system or method listed is relevant to your study. If you are not sure if a list item applies to your research, read the appropriate section before selecting a response.

Materials & experimental systems

n/a	Involved in the study
☒	☐ Antibodies
☒	☐ Eukaryotic cell lines
☒	☐ Palaeontology and archaeology
☒	☐ Animals and other organisms
☒	☐ Clinical data
☒	☐ Dual use research of concern
☒	☐ Plants

Methods

n/a	Involved in the study
☒	☐ ChIP-seq
☒	☐ Flow cytometry
☒	☐ MRI-based neuroimaging

Plants

Seed stocks	*Report on the source of all seed stocks or other plant material used. If applicable, state the seed stock centre and catalogue number. If plant specimens were collected from the field, describe the collection location, date and sampling procedures.*
Novel plant genotypes	*Describe the methods by which all novel plant genotypes were produced. This includes those generated by transgenic approaches, gene editing, chemical/radiation-based mutagenesis and hybridization. For transgenic lines, describe the transformation method, the number of independent lines analyzed and the generation upon which experiments were performed. For gene-edited lines, describe the editor used, the endogenous sequence targeted for editing, the targeting guide RNA sequence (if applicable) and how the editor was applied.*
Authentication	*Describe any authentication procedures for each seed stock used or novel genotype generated. Describe any experiments used to assess the effect of a mutation and, where applicable, how potential secondary effects (e.g. second site T-DNA insertions, mosiacism, off-target gene editing) were examined.*
